# Does the Establishment of National New Areas Improve Urban Ecological Efficiency? Empirical Evidence Based on Staggered DID Model

**DOI:** 10.3390/ijerph192013623

**Published:** 2022-10-20

**Authors:** Jingbin Wang, Huiling Qiao, Jing Liu, Bo Li

**Affiliations:** School of Management, Tianjin University of Technology, Tianjin 300384, China

**Keywords:** national new area, urban ecological efficiency, staggered DID model, urbanization level, urban transportation infrastructure

## Abstract

The environmental effects of national new areas have been an important topic but received little attention in academia. This study conducts a quasi-natural experiment using panel data of China’s 282 prefecture-level cities from 2006 to 2019, and evaluates the establishment of national new areas on urban ecological efficiency using the staggered difference-in-difference (DID) method, tests the robustness, and further examines the influential mechanism and urban heterogeneity of the empirical results. The results show that the establishment of national new areas has significantly improved urban eco-efficiency. Moreover, the mechanism analysis of the influences shows that national new areas improve urban eco-efficiency by improving urbanization level and urban transportation infrastructure. In addition, the heterogeneity analysis of cities shows that national new areas of cities in eastern and central regions are both significantly improving urban eco-efficiency, while those in western and northeastern regions are not. Furthermore, the promotion effect in the regions of “one new area in one city” model is better than that in “one new area in two cities” model; national new areas in non-resource-based cities show more positive effects on promoting urban eco-efficiency than those in resource-based cities. The conclusions reliably evaluate the results of the current construction of national new areas and provide feasible suggestions for further implementation of the related policy to balance economic development and environmental protection.

## 1. Introduction

As the world economy develops into a new stage, environmental problems, especially air pollution and climate warming, have become increasingly serious with the rapid economic growth. How to balance economic development and environmental protection is particularly urgent in developing countries [1]. Ecological efficiency (eco-efficiency) is often regarded as the lowest resource, with environmental input to achieve the maximum economic output [2]. An eco-efficient economy could reduce ecological damages to the minimum while maximizing economic efficiency [3], which reflects the sustainable ability of the region [4,5,6]. Numerous studies have investigated that rapid urbanization has posed a major threat to the ecological environment [7,8]. Therefore, focusing on urban construction to improve eco-efficiency is a starting point for integrating economic and environmental goals [9]. As the largest developing country in the world, the production mode of high input, consumption, and emission adopted is seriously threatening the sustainable development of China [10,11]. Therefore, it is necessary to explore the coupling coordination relationship between development construction and ecological environment through economic policies implementation to provide decision-making information for promoting urbanization construction and ecological environment protection.

It is apparent that improving urban eco-efficiency is inseparable from urban construction [12]. Since China’s reform, the country has experienced profound state rescaling that transforms city-regions to accommodate its exponential growth [13,14]. National new areas, as specific urban territories designated by the central government to undertake national strategic developmental and reform activities [15], serve regional prosperous strategies in recent years [16]. 19 new areas have been established in China until 2019. Prior research has investigated the economic performance implications of national new areas [17], yet the environmental effect of the policy implementation remains under researched. In fact, in the process of establishing national new areas, these areas have enhanced economic vitality, also suffered from a series of unprecedented ecological environmental challenges [18]. Therefore, exploring the environmental effect of the establishment of national new areas is of great significance for better realizing the whole benefits, and further strongly promotes national-new areas in ecological protection and high-quality development.

In this context, the purpose of this study is to investigate the environmental effect of the establishment of national new areas on urban eco-efficiency, the exact mechanism of that linkage, and the differences among different regions, providing scientific evidence to support the deep exploration of the policy effects of national new areas. The study area of this paper includes 282 cities in China from 2006 to 2019. Firstly, we collect different indicators of the input and output to construct the measurement system of urban eco-efficiency. The super-SBM model is proposed to calculate eco-efficiency in each city. Then, the environmental effect of the establishment of national new areas is tested using the staggered DID method. Next, this paper adopts a series of robust and endogeneity tests, such as the PSM-DID method and a placebo test, to verify the robustness of basic regression results. We especially draw on existing urban eco-efficiency literature to develop a research model that examines the mediating role of the urbanization level in delivering the commonly expected environmental benefits of national new areas. Finally, this paper introduces the influences of heterogeneity analysis among different regions. Based on the empirical results, we put forward corresponding measures for the coordinated development of the construction of national new areas and environment protection, and improving the quality of urbanization development from the perspective of single city. 

This research contributes to the existing literature in multiple ways. First, in terms of research theme, it adopts the DID model to provide a richer, more extensive conceptualization of environmental effects of national new areas that differ from other studies. This focuses on the economic effects of national new areas through multiple liner regression models, thus producing more comprehensive empirical results to test the whole effects of national new areas. Second, regarding the research content, based on the above results of national new areas, this paper further extends mediating effect by providing urbanization literature for the validity of the relationship between national new areas and urban eco-efficiency. The mechanism research contributes to specific routes to explain the relationship, and are of great practical significance to show how China can achieve sustainability and high-quality development. Third, the research discusses the heterogeneity analysis of different regions, areas distribution, geographic location, etc. to understand the detailed environmental effects of national new areas on urban eco-efficiency. The research results contribute to achieving coordinated development of the economy and ecology.

The remaining parts of this paper are divided into following sections: Section 2 is the literature review. Section 3 comprises the political background and research assumptions. Section 4 describes the research data, methods, variable selection, and data sources. Section 5 offers the empirical results and discussion, and Section 6 summarizes the research conclusions and puts forward corresponding policy implications. 

## 2. Literature Review

In recent years, the launch of national new areas has garnered widespread scholarly attention to explore the urban restructuring and effects of policy implementation [16,19]. Numerous studies focus more on the economic effects [20,21,22], urban rescaling [23,24], and industrial upgrading effects [25] of national new areas. Each new area represents the central government’s attempts to respond to the mounting social and environmental challenges generated by the rapid economic growth and excessive land development since economic reform [16]. Tientao (2016) provides a structural approach to verify that the establishment of national new areas significantly impacts productivity growth. In addition, limited by the number of research samples, there were several discussions on national new areas in the early stage, and most focused on analyzing and comparing the similarities and differences of existing urban structure [24]. However, the policy effects of studies on the relationship between the national new area construction and urban eco-efficiency still have some limitations and need to be further deepened and expanded. In fact, national new areas not only improve regional economic development, but also affect the urban industrial organization and ecological environment [26,27].

Ecological efficiency (eco-efficiency) reflects a coordination between economy and ecology, which is an important indicator for measuring the level of sustainable development and an important tool for management [28]. Existing studies have extensively explored the definition, influencing factors and measurement methods of urban eco-efficiency. Firstly, regarding to the meaning, eco-efficiency is often perceived as a win-win strategy to arrive at minor exploitation of natural resources and environmental impacts to create economic value [29]. Secondly, several researchers who have explored eco-efficiency’s influencing factors analyzed the impacts of GDP [30], technological progress [31], urbanization [32], industrial structure [33], environmental regulations [34], and other factors on eco-efficiency. Thirdly, accurate assessment of urban eco-efficiency is a prerequisite for urban sustainable development [35]. Summarizing the relevant literature, we observe that there are three main methods of measuring eco-efficiency, including the indicator system method [36], life-cycle assessment [37], and data envelopment analysis (DEA) [38]. It can be perceived that the DEA method is the most common method for measuring eco-efficiency. Super-SBM is used in this paper to assess the urban eco-efficiency, which incorporates undesired outputs into the evaluation of the relative effectiveness of DEA [39]. Therefore, determining the impact mechanism of urban eco-efficiency and studying the policy effects of national new areas on urban eco-efficiency is of great significance for policymakers to realizing sustainable urban development [40]. 

The relationship between regional eco-efficiency and socioeconomic or environmental performance has been laid an important foundation for understanding the national new areas and urban eco-efficiency, which provides a theoretical basis for this study. The establishment and construction of national new area is an effective measure to promote the rapid development of regional economics and accelerate the urbanization and modernization [41]. In the process of that, high-quality capital and labor factors are clustered to further complete urban construction and improve economic benefits [42]. Therefore, urbanization level increases gradually with the continuous optimization and improvement of industrial structure [43]. At the macro level, urbanization is considered the key factor affecting regional eco-efficiency. Some researchers have explained the mechanism of the two, believing that industrial agglomeration, population agglomeration, technology promotion, and lifestyle changes caused by urbanization have certain positive or negative effects on eco-efficiency [44]. Thus, the mediating role of urbanization needs to be further explored in the relationship between national new areas and urban eco-efficiency. In addition, construction of national new areas is beneficial to attracting industrial agglomeration, and enhancing business environments, such as urban transportation infrastructure [45]. Several empirical results show that optimizing urban transportation infrastructure may contribute to urban efficiency by the improvement of energy conservation, emission reduction, and efficiency promotion [46]. In summary, existing studies are insufficient to explain some of the existing problems and policy effects, so the urbanization level and transportation infrastructure should be considered as the mediating factor in the relationship between national new areas and urban eco-efficiency.

In summary, researches on urban eco-efficiency mainly focus on influencing factors and measurement methodology, and less attention has been paid to the effects of a certain development policy. Although the methodology and research framework have been formed around the research results, there is still room for improvement. Firstly, regarding the research perspective, the existing literature mainly focuses eco-efficiency on urban agglomerations [12,47] or provincial-level [48,49], and the research from prefecture-level cities perspective is scarce. Secondly, regarding the theoretical analysis, existing literature mainly focuses on economic development and industrial upgrading brought by the establishment of national new areas [50], and the researches rarely involves the environmental effects and eco-efficiency of national new areas. Thirdly, regarding to research methods, most studies investigate the exact mechanism between urban economic development and urban eco-efficiency mostly through multiple liner regression is adopted [51], and rarely research using the DID model to test the environmental effect of a certain urban policy.

## 3. Research Design, Data and Variables

### 3.1. Policy Background

As a comprehensive functional area approved by The State Council, national new areas undertake major strategic tasks of national development, reform, and opening up. They have a high level of autonomy and management advantages, and show significant influence on the economic development of located regions [17]. In 1992, China officially approved the establishment of first national new area—Shanghai Pudong new area—which began to explore the development path of national new areas. In April 2015, the NDRCPRC (National Development and Reform Commission of People’s Republic of China) issued the Guidelines on promoting healthy development of national new areas. National new areas in this report should focus on the comprehensive function and need to play an important role in promoting economic development, reform, and innovation. According to the report on the *Business Environment of China’s National New Areas 2018*, the GDP of top 18 national new areas in China has approached 4 trillion yuan, accounting for 5% of the whole GDP in 2017 [52]. Among them, GDP of 11 national new areas, such as the Shanghai Pudong new area, has reached 100 billion yuan. The results demonstrate that national new areas are gradually developing into a powerful engine of regional economic development. By the end of 2020, China has generally formed the basic pattern of “8 eastern + 2 central + 6 western + 3 Northeastern” of national new areas. The distribution and development stages of national new areas are shown in Table 1 below.

### 3.2. Research Assumptions

The national new area strategies are significant breakthroughs for national territory development and state spatial evolution [21]. According to recent theories in new economic geography [53] and relevant empirical studies, the full realization of the socioeconomic effect of the national new areas can be divided into the following two aspects: economic effects and environmental effects. On one hand, fostering the core, i.e., national new areas take the lead in forming an enterprise agglomeration and promote its rapid economic growth [54]. On other hand, the positive effect of national new areas on economic development is all-around and its effect on urban eco-efficiency cannot be ignored.

From the perspective of absorbing factor agglomeration, national new areas are approved and established by the State Council, which reflect national strategic needs. Therefore, a higher administrative degree is conducive to attracting more factor agglomeration [55], improving the production efficiency of enterprises, reducing energy consumption and pollution emission per unit output, and thus improving urban eco-efficiency [56]. The research showed that, on the premise of ensuring enterprise demand and improving productivity, manufacturing agglomeration can mitigate the impact of “pollution paradise” effect [57]. 

From the perspective of realistic development needs, national new areas have the “green ecology” demand of building green ecological and livable new urban areas [58]. With the agglomeration of capital, high technology and other factors, production technological innovation can be accelerated to reduce pollution emission of enterprises. At the same time, environmental protection regulations guide more output to be allocated to high-productivity enterprises, which is conducive to the optimization and redistribution of resources among enterprises, and ultimately improves eco-efficiency [59]. Therefore, some researchers have confirmed that development zones play an irreplaceable role in promoting industrial agglomeration and structural upgrading, improving the efficiency of resource allocation, promoting employment and economic growth, and improving regional environmental performance at the macro-level [60]. Thus, Hypothesis 1 is proposed as follows:

**Hypothesis** **1:** *The establishment of national new areas is conducive to improving urban eco-efficiency*.

National new areas, as the frontier of modern industrial agglomeration, lead a new direction of urbanization [61]. Generally, the establishment of national new areas is conducive to improve regional economic growth [62]. Some recent studies have empirically tested national new areas in a spatial agglomeration of economic activities, accelerating the urbanization and industrialization process in China [63]. Establishing the national new area is found to have increased the spatial inequality of the municipality where it is located, and the level of the regional economy, degree of openness, and complexity of administration has identified to be affected [13]. As the critical construction project for regional economic development, national new areas are authorized preferential policies in credit and land usage, and attract high quality resources, such as labor, capital, and information [17], so the urbanization level improves with it [64]. Meanwhile, the improvement of urbanization level is conducive to the upgrading of industrial structure and transformation of urban lifestyles. Several studies have explained the mechanism of urbanization affecting eco-efficiency by considering intermediate variables [65], believing that industrial agglomeration, population agglomeration, technology promotion, and lifestyle changes caused by urbanization have certain positive or negative effects on eco-efficiency. 

Besides the urbanization, urban transportation infrastructure plays an important role in the research of national new areas and urban eco-efficiency. The establishment of the national new areas would inevitably lead to the optimization of local transportation infrastructure and management system [17]. Moreover, an important factor about the ecological efficiency relates urban traffic conditions. Some scholars have reported that urban transportation infrastructure, such as broadband infrastructure, can reduce production and transaction costs [66], promote the efficiency of green transformation [67], and be an essential driving force for improving urban eco-efficiency [68]. Therefore, it can be clear that urban transformation infrastructure is gradually completed, driven by the process of national new area construction. In conclusion, both urbanization level and urban transportation infrastructure could be considered important mediating factors in the research of exploring the environmental effect of national new areas on urban eco-efficiency, which can accelerate the transformation of development mode and improve urban eco-efficiency. Thus, Hypothesis 2a and Hypothesis 2b are proposed as follows:

**Hypothesis** **2a:** *The establishment of national new areas promotes urban eco-efficiency by improving urbanization levels.*

**Hypothesis** **2b:** *The establishment of national new areas promotes urban eco-efficiency by completing urban transportation infrastructure*.

### 3.3. Model

Based on the research hypotheses and existing empirical literature [69], we can verify the policy effects of national new areas established on urban eco-efficiency. This paper determines whether the establishment of national new areas is regarded as a quasi-natural experiment, and a staggered DID model is used to test the policy effectiveness. Since national new areas are established in batches, the staggered DID (difference-in-differences) model is constructed, which is as follows:

(1) $$EE_{it}=\alpha_{0}+\alpha_{1}{did}_{it}+\sum_{j}\beta_{j}Control_{it}+\gamma_{t}+\eta_{i}+\varepsilon_{it}$$

where *i* denotes the city and *t* denotes the year. *EE_it_* represents the eco-efficiency of city *i* in year *t*, which is the core explanatory variable. *did_it_* represents whether city *i* establish national new areas or not in year *t*, so *did_it_* = 1 when the city is approved to establish national new areas in the year and subsequent years, and *did_it_* = 0 when the city is not established national new areas. *Control_it_* represents other control variables in the model that may affect firm urban eco-efficiency which changes as year and city. *γ*_t_ represents time fixed effects, and *η*_i_ represents individual fixed effects. *ε*_it_ is a random error term. 

### 3.4. Variable Description

(1) Explained variable is the urban eco-efficiency *EE_it_*. The index of eco-efficiency is the ratio of economic growth and environmental resource consumption [70]. Data envelopment analysis model (DEA) is used by most researchers to calculate eco-efficiency of each city, but this method cannot distinguish the efficiency of existed decision-making units (the efficiency value reaches 1). Therefore, based on the input and output perspective, this paper uses super-SBM model proposed to measure eco-efficiency in each city in 2006–2019, and the indicators include input factors, desire outputs, and undesired outputs.

The inputs include labor, capital, land, and energy resources, with labor resources input being measured by the number of employees in each city at the end of year, while the capital input is the capital stock of each city. The perpetual inventory method is used to calculate with 2006 as the base period. The ideal index of land resources input is the construction land area. The index of total electricity consumption is adopted as energy input.

The desirable outputs include economic development, fiscal revenue, and urban landscaping, which is consistent with the majority of scholars. Regional GDP was selected for measurement and the influence of the price factor was removed. Fiscal revenue is represented by local budgetary revenue and calculated with 2006 as the base period. Urban landscaping is greenery coverage of an urban area. The undesirable outputs are industrial discharged wastewater, industrial sulfur dioxide emission, and industrial soot (dust) emission, and are based on the availability of data. The input-output indicators and calculations are shown in the Table 2 below:

(2) Core explanatory variable. *did_it_* reflects the establishment of national new areas is the interaction term, i.e., *did_it_* = *treat_i_* × *post_t_*. Of these, *treat_i_* is the dummy variable that distinguishes the treatment group from control group, *post_t_* is the dummy variable that distinguishes before and after treatment. If city *i* is approved to establish a national new area during the sample period, it belongs to the treatment group, *treat_i_* = 1; otherwise, it belongs to the control group, *treat_i_* = 0. If city *i* is approved to establish a national new area in year *t* of the sample period, then *post_t_* = 1 from the beginning of the year, otherwise, *post_t_* = 0. Therefore, in the sample period, *did* = 1 in the year when a city was approved to establish a national new area and the year after, otherwise, *did* = 0.
(3) Control variables. Considering that many factors other than the policy of national new areas will affect urban eco-efficiency, this paper selects control variables from five aspects of industrial structure, innovation, openness, scientific and technological investment, and government scale. Specifically, advanced industrial structure is measured by the following [71]:
  (2) $$\theta_{j}=arccosf\left(\frac{\sum_{i=1}^{3}\left(x_{i,j}\cdot x_{i,0}\right)}{{\left(\sum_{i=1}^{3}x_{i,j}^{2}\right)}^{\frac{1}{2}}\cdot {\left(\sum_{i=1}^{3}x_{i,0}^{2}\right)}^{\frac{1}{2}}}\right)\;$$
  (3) $$W=\sum_{k=1}^{3}\sum_{j=1}^{k}\theta_{j}$$
  where *j* = 1,2,3, and the larger the *W*, the higher the advanced industrial structure; the innovation level is represented by the number of granted patents; level of openness adopts the ratio of actual utilized foreign capital to regional GDP to measure [72]; the level of science and technology investment is measured by the ratio of science expenditure to GDP [73]; government scale is measured by the proportion of government spending in regional GDP [74].
(4) Mediating variables: one is urbanization level. In this paper, the proportion of urban non-agricultural population in the total population is used to measure the urbanization level [75]. The detailed employment data in the specific industry in this part are mainly obtained from 2007–2020 from China City Statistical Yearbook. Missing data are supplemented by interpolation. The other is urban transportation infrastructure, which is measured by highway freight traffic [76]. The index is the key indicator to reflect the transportation accessibility and convenience of the city. The detailed data comes from China City Statistical Yearbook. Table 3 is the descriptive statistics of variables in this paper:

### 3.5. Data Selection

Data for labor, energy consumption, and other variables are obtained from 2006–2019 from China City Statistical Yearbook, China Statistical Yearbook for Regional Economy, China Energy Statistical Yearbook, China Environment Yearbook, Statistical Bulletin of Chinese Provinces and Cities, and China Research Data Service platform (CNRDS). At the same time, considering the availability of data, balanced panel data of 282 Cities in China from 2006 to 2019 were selected for analysis, and the missing values were supplemented by linear interpolation method.

## 4. Empirical Research

This paper evaluates environmental effects of national new areas on urban eco-efficiency, which is divided into the following parts: Firstly, the effects of national new areas on urban eco-efficiency are measured by using the staggered DID model. Secondly, a robustness test of the empirical results obtained in the first step is carried out. Thirdly, heterogeneity analysis of policy implementation is further discussed. Fourthly, the influential mechanism of national new areas on urban eco-efficiency is preliminarily analyzed.

### 4.1. Parallel Trend Assumption Analysis

**Figure 1:**
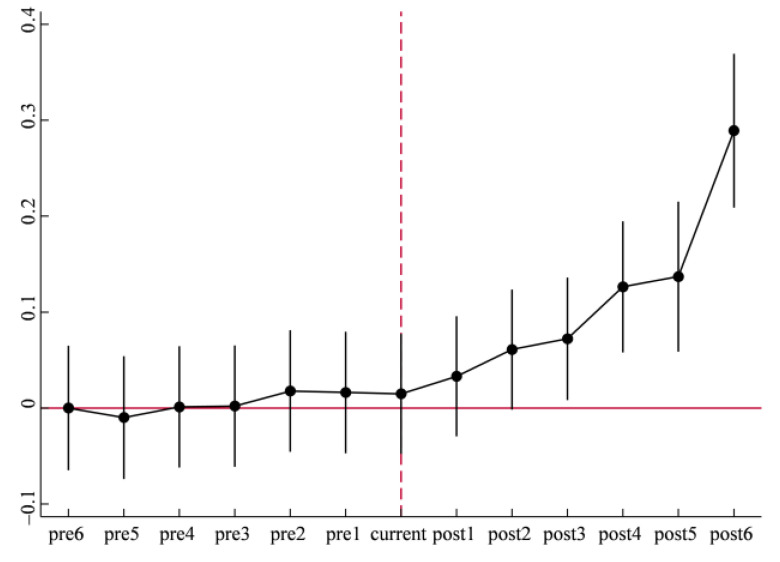
Parallel trend testing.

The prerequisite for policy evaluation using the difference in differences (DID) method is that the treatment group and control group have the same trend before implementation of policy, that is, parallel trend assumption is required. Therefore, to evaluate policy effect of national new areas, it is essential to conduct a parallel trend assumption on the explained variable: eco-efficiency (*EE*) of each city, i.e., to test whether there is significant difference of carbon emission efficiency between the city establishing national new areas and other cities before establishing. Learning from Beck et al., 2010, the event study method is adopted to analyze the change in trend of treatment group and control group. If there are no apparent differences between the two, the parallel trend test is accepted. The model are as follows [77]:

(4) $$EE_{it}=\alpha_{0}+\alpha_{1}{\mathrm{did}}_{it}^{-6}+\alpha_{2}{\mathrm{did}}_{it}^{-5}+\ldots +\alpha_{13}{\mathrm{did}}_{it}^{6}+\underset{j}{\sum }\beta_{j}Control_{it}+\gamma_{t}+\eta_{i}+\varepsilon_{it}$$

where *did_it_*^±*j*^ is a series of policy dummy variables, and the rule of selecting values is that *did_it_*^−*j*^ (*did_it_^j^*) is 1 when *j* year before (after) cities of treatment group is established national new areas, otherwise it is 0, and the meanings of other variables are the same as the model (1). In addition, ${\mathrm{did}}_{it}^{6}$ represents cities of treatment groups that have established national new areas for 4 years or more. Figure 1 shows the results of model (4), indicating that the 95% confidence interval of regression *α*_1_, …, *α*_6_ all contain 0, so the regression results are not significant, which indicates that there is no significant difference between the treatment group and control group, and it passes the parallel trend assumption. After the implementation of the policy, 95% confidence intervals of the regression coefficient *α*_10_, …, *α*_13_. are all greater than 0, and the value of coefficient gradually increases in fluctuation after the policy implementation. These results show that the establishment of national new areas increases the urban eco-efficiency, and the promotion effect will gradually expand as time goes by. 

### 4.2. Benchmark Analysis

The research described in this paper uses stata15.0 software to conduct empirical tests. Stata is a statistical analysis system of a company in the United States to conduct some empirical studies. First, we estimate the direct influence of national new areas to urban eco-efficiency to verify H1. Table 4 reports the results of Equation (1). Columns (1) and (2) of Table 4 show that the coefficient of core explanatory variable *did* is significantly positive with or without control variables at 1% level, implying that the establishment of national new areas is beneficial to improve urban eco-efficiency, verifying H1 greatly. Therefore, national new areas have great potential in improving urban ecological environment, which is consistent with previous theoretical results [53]. This occurs because the establishment of national new areas accelerates the agglomeration of factors, reduces unit energy consumption, and further improves urban eco-efficiency. 

### 4.3. Robustness Analysis

#### 4.3.1. Regression Analysis Based on Propensity Score Matching and the Dual Difference Model (PSM-DID)

The PSM-DID method is adopted to re-evaluate the policy effects of national new areas on urban eco-efficiency, so as to confirm whether the conclusion is still robust after correcting data selection bias. Therefore, firstly, PSM method is used, and control variables in the benchmark model are adopted to predict the probability of establishing the national new areas in each city through using Logit model. Secondly, the nearest neighbor matching method is used for sampling [63] what is put back, and then treatment and control groups are matched one-to-one. Finally, the mean difference between rematched treatment and control groups is examined, and the “pure effect” of policy implementation is assessed using the staggered DID method. Considering that the samples after PSM are also required to pass the parallel trend assumption, this paper further conducted parallel trend testing again after matched samples.

**Figure 2:**
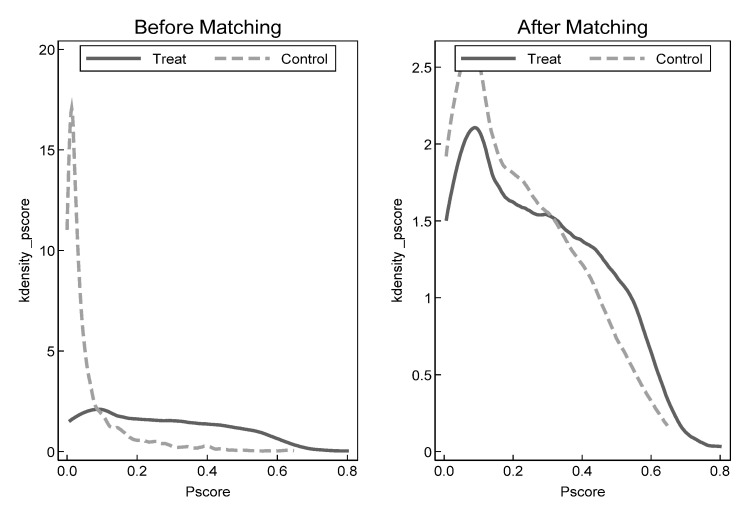
Comparison of propensity score distribution between treatment group and control group before and after PSM.

**Figure 3:**
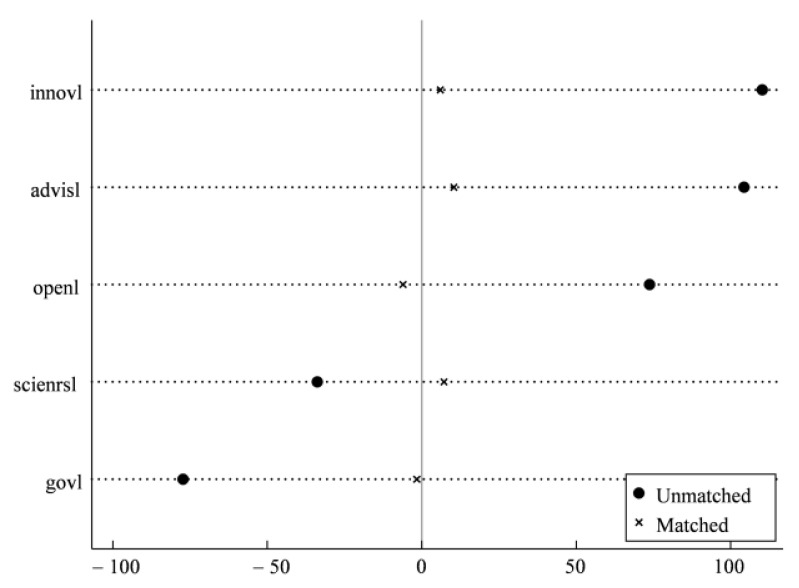
Standardized % bias across covariates.

**Figure 4:**
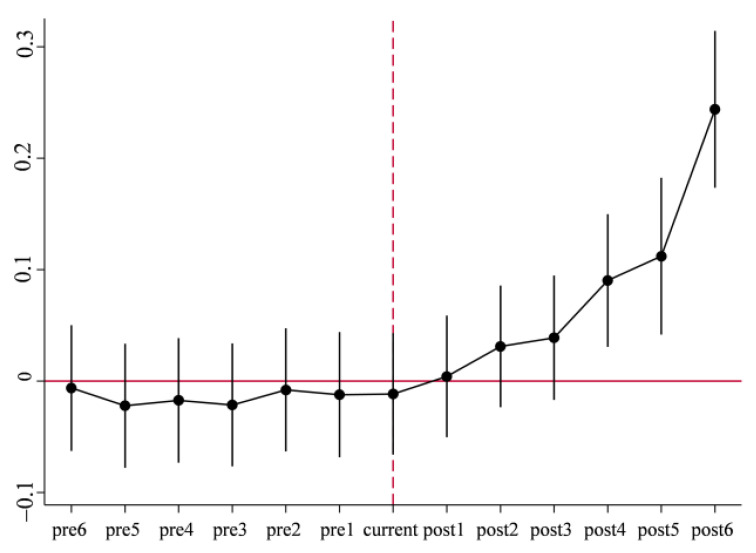
Parallel trend testing after PSM.

Figure 2 shows the density distributions of propensity scores in the treatment and control groups before and after matching. It can be seen that distributions of propensity scores in two groups are basically the same, and the initial characteristics of two groups samples are similar. Meanwhile, Table 5 reports the balance test results of matching using city-level control variables as covariates, which indicate that *t* values of the covariates are all not significant after matching. There exists no significant differences between the treatment group and control group. In addition, it can be seen when combined with Figure 3 that the standardized deviations of each variable are closer to 0, and most of them are less than 10% after matching, indicating that the deviation of covariates of two groups is effectively reduced because of matching, that is, the covariates of treatment group and control group are basically balanced after matching. There still exists no significant difference between treatment group and control group after excluding the unmatched samples through PSM, which passes the parallel trend testing. Based on balance testing and parallel trend testing, the benchmark regression on matched new samples is conducted in this paper, and the estimation results in columns (3) and (4) of Table 4 above show that the coefficients of core explanatory variable *did* are all significant and close to benchmark regression results, which indicates the robustness of regression results. In addition, some unmatched samples are excluded using propensity score matching and new matched samples are used again to conduct parallel trend testing in Figure 4. The results are consistent with above results, and effectively solves the apparent differences among samples.

#### 4.3.2. Placebo Test

It is considered that the influence of national new areas on urban eco-efficiency may be caused by some unobserved systematic differences as time goes on, rather than direct influence brought by implementation of the policy. Therefore, the placebo test is conducted in this paper to further guarantee the reliability and robustness of results. The time period of this paper is from 2006 to 2019, so the establishment of national new areas during this period will have an influence on urban eco-efficiency after 2019, but it will not have an obvious influence before 2006. The national new areas establishing the year of each city is assumed to be before 2006, and constructs “virtual national new areas establishment” in three periods, that is, namely 3 years, 5 years, and 7 years in advance, respectively.

Columns (1), (2), and (3) in Table 6 introduce the results of placebo test, which indicate that the establishment of national new areas three years ahead of the actual situation, which still contributes to the improvement of urban eco-efficiency at 5% level. The reason is that the national new areas are mostly constructed several years in advance, and the related projects of each city have also been started. Therefore, the positive effects of national new areas in promoting urban eco-efficiency have begun to emerge. However, the coefficients of national new areas are no longer a significant 5 and 7 years ahead of actual situation, and coefficients are gradually decreasing, which indicates that it is obvious that positive effect of national new areas on urban eco-efficiency are obvious in a limited time, and the placebo test results show that there exists the most significant positive effect of national new areas on urban eco-efficiency in the research period of this paper, which further supports the conclusions above.

#### 4.3.3. Change of Sample Period

In order to verify the robustness of benchmark regression results and avoid the effects of other factors, such as the global financial crisis in 2008 and natural disasters on the policy results, this paper further selects the sample period from 2009 to 2018 to analyze the regression again. Column (4) of Table 6 reports the results which indicate that the coefficient of *did* increases, but the direction and significance level are consistent with the benchmark regression results in Table 4. These results show that the establishment of national new areas still shows the significant positive effect on urban eco-efficiency after a global financial crisis, which further verifies the robustness of above results.

#### 4.3.4. Change of Lag Period of Control Variables 

Considering that there may be reverse causality between each control variable and the establishment of national new areas, this may lead to endogeneity problems in the model. This paper re-established the staggered DID model for regression by using the control variables with lag 1 period and lag 2 period, respectively. The estimation results are shown in column (5) and column (6) in Table 6. The results are consistent with Table 4, verifying the robustness of mentioned conclusions.

#### 4.3.5. Excluding the Effects of Other Policies

In order to exclude the effects of other policies on urban eco-efficiency in the same period and test whether the effect of national new areas on urban eco-efficiency is the “pure effect” in benchmark regression, meaning the effect is not be affected by other policies, this paper selects the policy of “national high-tech zones”, which are similar to national new areas, to be included in the regression. The results in column (7) of Table 5 show that the effect of national new areas on urban eco-efficiency is still significant at 1% level and the coefficient increases after the policy of “national high-tech zones” added. That is, the establishment of national new areas exactly improves urban eco-efficiency, which is consistent with the benchmark regression. The above results are further verified. 

### 4.4. Mechanism Analysis

From the above empirical analysis results, we can see that the establishment of national new areas significantly improve urban eco-efficiency. Combined with the above analysis and H2, in order to recognize the mechanism of the two, mediation model is used to further test. Specifically, based on model (1), the following recursive model is constructed:

(5) $$EE_{it}=\alpha_{0}+\alpha_{1}{\mathrm{did}}_{it}+\underset{j}{\sum }\beta_{j}Control_{it}+\gamma_{t}+\eta_{i}+\varepsilon_{it}$$

(6) $${\mathrm{urbanl}}_{it}/glhy_{it}=\varphi_{0}+\varphi_{1}{\mathrm{did}}_{it}+\underset{j}{\sum }\beta_{j}Control_{it}+\gamma_{t}+\eta_{i}+\varepsilon_{it}$$

(7) $$EE_{it}=\lambda_{0}+\lambda_{1}{\mathrm{did}}_{it}+\lambda_{2}{\mathrm{urbanl}}_{it}/glhy_{it}+\underset{j}{\sum }\beta_{j}Control_{it}+\gamma_{t}+\eta_{i}+\varepsilon_{it}$$

where *urbanl_it_* represents mediating variable-urbanization level, *glhy_it_* represents intermediate variable-highway freight traffic, and meanings of other variables are the same with model (1). The test of mediation model is divided into three steps. Firstly, estimate coefficient *α_1_* of model (5). It is necessary to test total effect of national new areas on urban eco-efficiency. If *α_1_* is significantly positive, it means the establishment of national new areas could improve urban eco-efficiency. Secondly, estimate coefficients *φ_1_* and *λ_2_* of model (6) and (7) separately. If the two are significant, it indicates the existence of mediating effect. Based on this, *λ*_1_ is not significant, meaning that the mediating variable is full mediation; *λ_1_* is significant, positive, and smaller than *α*_1,_ which means the mediating variable is partial mediation. Finally, if at least one of the coefficients *φ*_1_ and *λ*_2_ is not significant, the Sobel test will be used to judge whether the mediation effect exists or not. The estimated results are reported in Table 7. 

It can be seen from Table 7 that: firstly, column (1) shows that the establishment of national new areas could significantly improve urban eco-efficiency; secondly, columns (2) and (4) shows that coefficient *φ*_1_ of *did* in model (6) is significantly positive, and columns (3) and (5) represents coefficient *λ*_2_ of *urbanl_it_* or *glhy_it_* in model (7) is significantly positive at the 1% level, and coefficient *λ*_1_ of *did_it_* in model (7) is also significantly positive at the 1% level. The results indicate that mediating variables i.e., urbanization level and urban transportation infrastructure both play the partial intermediary role in the total effect. Further calculation finds that the mediation effect of urbanization level and urban transportation infrastructure accounted for 5.42% and 1.92% of total effect, separately. The above analysis implies that national new areas significantly promotes urban eco-efficiency by promoting urbanization level and urban transportation infrastructure. A potential reason is that national new areas’ construction is significantly and inevitably to attracting high-quality resources and talents to complete urbanization construction, promoting the continuous improvement of high-quality transportation infrastructure, and enabling lower energy consumption per unit output, thus enhancing eco-efficiency [78]. Therefore, both theoretical and empirical analysis verified the reasonableness of H2a and H2b.

### 4.5. Heterogeneity Test

#### 4.5.1. Regional Heterogeneity Test

(1) Heterogeneity test of location area. Considering that the industrial structure is relatively more reasonable, the level of economic marketization and openness is relatively high, and the policy implementation environment is relatively good in eastern and central regions of China, economic development in western and northeastern regions are relatively falling behind due to history, location, resource endowment and other factors, so the environment for policy implementation is relatively poor. In order to further investigate whether there are significant differences in the effects of national new areas on urban eco-efficiency in different regions, the sample cities in this paper are divided for heterogeneity test, and the estimated results are reported in Table 8.

It can be seen from the first four columns of Table 8 that the differences in economic development in the early stage will lead to different policy effectiveness of national new areas in different regions. Of that, national new areas of cities in eastern and central regions are both significantly improving urban eco-efficiency, while the cities in western and northeastern regions are not. One important reason is that due to the superior geographical location, the economic development of eastern and central regions is faster than that of other regions, so national new areas in these cities are most significant to improving the urban eco-efficiency. Economic development and other aspects of the above areas have been relatively mature, which puts a solid foundation for the establishment of national new areas. The results are consistent with existing studies, which show that the improvement of technology-intensive industries agglomeration can reduce resource consumption and environmental disruption, and thus improve urban eco-efficiency [79]. On the contrary, western region is relatively falling behind, and ecological environment is relatively fragile, so the policy effect of national new areas in the western region is insignificant to urban eco-efficiency. As a traditional industrial basement in China, northeastern region suffers from severe ecological and environmental problems caused by overexploitation and utilization of resources. In addition, the market development and institutional construction are relatively falling behind, so that national new areas have no significant influence on urban eco-efficiency in this region.

(2) Heterogeneity test of coastal or inland. In order to further verify whether the establishment of national new areas could effectively promote the coordinated development of coastal and inland areas, the cities are divided into coastal cities and inland cities according to location to further conduct the regression model separately. Columns (5) and (6) in Table 8 show that the coefficient of national new areas in coastal cities is significant positive at 1% level, but the coefficient of national new areas in inland cities is not significant, which indicates that policy effects are more positive of national new areas in coastal cities.

#### 4.5.2. Heterogeneity Test of Layout Model of National New Areas

The construction and development of national new areas are inseparable from the planning of administrative departments. Theoretically, the model of “one new area in one city” is conducive to the coordination among various departments, improving the administrative efficiency, giving full play to main advantages of the region, and thus promoting the formation of the “polarization effect”. However, the model of “one new area in two cities” is conducive to the information exchange between regions, strengthening the division of labor and cooperation between regions, and benefiting exertion of the “irradiation effect” [80]. Columns (1) and (2) in Table 9 report the regression results of different layouts of national new areas. The results show that the coefficient of *did* in column (1) of Table 9 is significant positive at 1% level, and that is not significant in column (2), which indicates that the promotional effect of the national new areas in “one new area in one city” model is better than that in “one new area in two cities”. The reason is maybe that national new areas in the layout of the “one new area in two cities” model not only produces regional synergistic growth effect, but also brings coordinated challenges within the city and pressure on resources, so environment protection has increased accordingly.

#### 4.5.3. Heterogeneity Test of Different Types of Cities

Resource-based cities are an indispensable and important part in the Chinese urban system, and rely on the coal mining and processing of non-renewable resources, such as minerals, as the main development path. The traditional development model, which lacks innovation, has led to slow development of high-quality development of resource-based cities. The related research of resource-based cities mostly focuses on influencing factors of industrial structure transformation. Few studies focus on how to improve the eco-efficiency of resource-based cities. Based on this, this paper further explores differences in the influence of national new areas on eco-efficiency of resource-based cities and non-resource-based cities in order to provide new ideas for transformation and development. The list of 116 resource-based cities is from National Sustainable Development Plan for Resource-based Cities (2013–2020). Bijie, Jinchang, and Laiwu are excluded in this paper due to data availability. 113 resource-based cities are finally retained. Columns (3) and (4) in Table 9 report the regression results of resource-based cities and other cities which are the coefficient of *did*. In column (4), it is significant positive at 1% level, but that in column (3) is not significant. These findings indicate that the establishment of national new areas in non-resource-based cities show more positive effects on promoting urban eco-efficiency than resource-based cities. The reason is that economic growth of most resource-based cities is slowing down; the ecological environment is seriously damaged, urban location is not good, and supporting infrastructure is not complete, which leads to the establishment of national new areas. Therefore, there cannot be timely improvement to regional economic development.

## 5. Discussion

In-depth study of environmental effects of national new areas and urban eco-efficiency related literature can help in mastering the knowledge of related fields, and can also play a reference role for our own research. As the establishment of national new areas could attract many high-quality industrial and talents resources, national new area construction is of great significance to improve urbanization level and optimize urban transportation infrastructure, which can effectively promote urban eco-efficiency and contribute to balancing economic development and environmental protection. In the part of literature, we focus on the origin and development of national new areas, urban eco-efficiency, and the influence mechanism of both, and put forward the views of this paper. National new areas are a comprehensive economic development plan. Compared with exploring economic effects of national new areas, there is little evidence on the environmental impact of that in previous studies in the context of China. This study exploited the staggered DID estimation to investigate the causal relationship between economic development area and urban eco-efficiency based on the China City Statistical Yearbook and other datasets. Moreover, we further explored the possible mechanisms and heterogeneous effects of economic development policy to detailed understand the environmental effects of urban eco-efficiency. 

In recent years, scholars have paid attention to national new areas’ contribution to the high-quality economic development and urban construction [17]. The development of China’s National New Areas has attracted the concentration of high-quality production factors, such as the talents, and transportation infrastructure, and could empower traditional industries, and improve resource utilization rate [81]. We conducted special research on the environmental effects, focusing our attention on the relationship between national new areas and urban efficiency. At present, the research on national new areas only mentions that establishment and construction of national new areas is helpful to social and economic development [17], and few authors directly discuss the environmental effects of national new areas on urban eco-efficiency. This paper discusses whether the establishment of national new areas could improve urban eco-efficiency from the macro-angle, enriching the research in related fields. In the process of establishment and construction of national new areas, driving urban eco-efficiency through increasing urbanization level and improve transportation infrastructure and realizing the coordination of environmental protection and economic development.

There are some limitations in this study. Future research will complement the shortcomings of this study and provide reference direction for policy research. While this study has analyzed the impact of national new areas on urban eco-efficiency and its exact mechanism, the analysis is not comprehensive owing to data limitations given the limited number of policy implementations. In the future, it is necessary to explore the long-term dynamic effects of the policy, further identify more mediators to explain the relationship between national new areas and eco-efficiency, and improve the accuracy of the conclusion. However, as policies with Chinese characteristics, policy effect is different from other developed other regions. Therefore, our future research needs to improve its universality in the world, providing helpful suggestions for balancing global economic development and ecological protection. 

Above all, this paper innovatively focuses on the certain policynational new areas to study the environmental effects of national new areas on urban eco-efficiency, which is a useful supplement to existing studies. Moreover, we also fully explore the performance of regional and new areas layouts’ heterogeneity in the relationship between the two, and test the moderating effect of urbanization level and urban transportation infrastructure, which provides a reference for policymakers to better balance economic development and environmental protection. As a result, our findings are helpful for the construction of an accurate policy package to alleviate the negative environmental effects of national new areas on urban efficiency.

## 6. Conclusions

Currently, not only for developed countries, but also developing countries like China, we attach importance to environmental changes brought about by economic development. Although there is certain environmental pressure of economic policy implementation in many ways, in terms of air pollution and climate change, our study finds a positive environmental effect of national new areas on urban eco-efficiency, which provides some reference for the policymakers. 

### 6.1. Main Findings 

Based on panel data of 282 prefecture-level cities in China from 2006 to 2019, this research empirically tested the effect of national new areas on urban eco-efficiency using a series of methods, including a staggered DID, parallel trends test, PSM test, and placebo test. The main conclusions of this study are: (1) the establishment of national new areas has significantly improved urban eco-efficiency. This result is consistent with previous study which presented evidence that national new area construction is beneficial to social progress [82]; (2) The finding further implies that the establishment of national new areas affect urban eco-efficiency not only through improved urbanization level, but also through enhanced transportation infrastructure; (3) The heterogeneity analysis shows that national new areas of cities in eastern and central regions both significantly improve urban eco-efficiency, while that in western and northeastern regions are not significant to urban eco-efficiency; moreover, the promotion effect of national new areas on urban eco-efficiency in the regions of “one new area in one city” model is better than that in “one new area in two cities” model; (4) Finally, the establishment of national new areas in non-resource-based cities show more positive effects on promoting urban eco-efficiency than that in resource-based cities.

### 6.2. Policy Implications

Combining the above conclusions, the policy implications are as follows. First, we should give full play to the effect of national new areas in improving ecological environment and enhancing eco-efficiency. The core finding demonstrates the establishment of national new areas and indeed plays a positive role in improving urban eco-efficiency. To further improve urban eco-efficiency, we should comprehensively consider the orientation, resource endowment and climatic conditions of each national new area, and focus on the environmental issues in the process of promoting economic development, so as to play a leading role in terms of economic development and ecological environmental protection. Second, regional differences should be paid attention to and regions should rationally distribute national new areas. The analysis of this paper shows that environmental effects of national new areas have obvious regional heterogeneity and indicates that the regional characteristics should be considered in the process of distribution of national new areas, such as giving strong policy support to solve the dilemma of insignificant promotion effects of national new areas in western region, summarizing the successful experience of “one new area in one city” mode, and exploring a new mode of national new areas. Finally, improving the business environment of the located city, and increasing the attractiveness and mobility of factor resources has its implications on policy. This paper shows that urbanization level and urban transportation infrastructure are mediating variables for national new areas to improve urban eco-efficiency. Therefore, the national new areas of located city should take make use of its institutional advantages to focus on developing differentiated layouts, enhancing the attractiveness of high-quality resources, improving urbanization level, and completing urban transportation infrastructure, so as to realize coordinated progress of economic development and environmental protection.

## Figures and Tables

**Table 1:** Regional Distribution of National new areas.

Regional Distribution	National New Areas
Eastern region	Shanghai Pudong new area Tianjin Binhai new area Zhoushan Qundao new area Guangzhou Nansha new area Qingdao Xihaian new area Nanjing Jiangbei new area Fujian Fuzhou new area Hebei Xiongan new area
Western region	Chongqing Liangjiang new area Gansu Lanzhou new area Guizhou Guian new area Sichuan Tianfu new area Yunnan Dianzhong new area Shaanxi Xixian new area
Central region	Hunan Xiangjiang new area Jiangxi Ganjiang new area
Northeastern region	Dalian Jinpu new area Harbin new area Changchun new area

Note: Data comes from the Chinese government website: www.gov.cn. accessed on 27 April 2019.

**Table 2:** Input-output index system of urban eco-efficiency.

Index	Variables	Unit	Calculation
Input	the number of employees	Ten thousands/people	
	Fixed capital stock	Ten thousands/yuan	$K_{it}=K_{it-1}(1-\delta_{it})+I_{it}$
	Construction land area	square kilometers	-
	Social power consumption	Ten thousands/kwh	-
Desirable Output	GDP	Ten thousands/yuan	-
	Fiscal revenue	Ten thousands/yuan	-
	Urban landscaping	%	-
Undesirable Output	Industrial sulfur dioxide emission		-
	Industrial discharged wastewater		-
	Industrial soot (dust) emission		-

Notes: *K_it_* is fixed capital stock of the current year; *I_it_* is fixed asset investment in the current year; *δ_it_* is the rate of eco nomic depreciation.

**Table 3:** Descriptive statistics of variables.

Variable Category	Variable Name	Variable Unit	Observation	Mean	Mean Standard	Minimum	Maximum
Explained variable	EE	-	3948	0.240	0.191	0.004	1.378
Explanatory variable	did	-	3948	0.0382	0.192	0	1
Intermediary variables	urbanl	%	3948	0.386	0.226	0.0755	1.819
	glhy	billion tons	3948	1.034	1.545	0.0213	55.4203
Control variables	advisl	-	3948	6.461	0.371	5.407	7.652
	innovl	-	3948	6.675	1.800	1.099	12.02
	openl	-	3948	0.0183	0.0193	0.000	0.199
	scienrsl	%	3948	0.00698	0.00742	0.000	0.118
	govl	%	3948	0.658	0.637	0.0468	5.852

**Table 4:** Benchmark regression results.

Variable	(1) *EE*	(2) *EE*	(3) *EE*	(4) *EE*
did	0.0726 ***	0.0621 ***	0.0558 ***	0.0497 ***
	(0.0152)	(0.0150)	(0.0134)	(0.0134)
advisl		−0.0100		−0.0002
		(0.0188)		(0.0180)
innovl		−0.0284 ***		−0.0036
		(0.0051)		(0.0055)
openl		−0.2950 *		−0.1019
		(0.1703)		(0.1559)
scienrsl		0.0566		−0.5006
		(0.4389)		(0.7976)
govl		−0.0776 ***		−0.0718 ***
		(0.0093)		(0.0132)
_cons	0.2228 ***	0.4649 ***	0.1977 ***	0.2421 **
	(0.0072)	(0.1187)	(0.0067)	(0.1162)
N	3948	3948	3449	3449
R^2^	0.155	0.182	0.238	0.247

Notes: Robust standard errors are reported in parentheses; * *p* < 0.1, ** *p* < 0.05, and *** *p* < 0.01, respectively.

**Table 5:** PSM and balance test results.

Variable	Coefficient	Sample	Mean		% Bias	t	*p* > │t│
			Treated	Control			
advisl	1.2157	Unmatched	6.7969	6.4308	104.3	17.62	0.0000
	(0.2472)	Matched	6.7939	6.7572	10.4	1.35	0.1780
innovl	0.5862	Unmatched	8.3727	6.5245	110.2	18.40	0.0000
	(0.0598)	Matched	8.3470	8.2467	6.0	0.79	0.4310
openl	19.3143	Unmatched	0.0327	0.0170	73.8	14.28	0.0000
	(2.7000)	Matched	0.0321	0.0334	−6.1	−0.61	0.5430
scienrsl	−181.7845	Unmatched	0.0051	0.0071	−33.8	−4.66	0.0000
	(21.0762)	Matched	0.0051	0.0047	7.2	1.53	0.1270
govl	0.0510	Unmatched	0.3123	0.6889	−77.3	−10.31	0.0000
	(0.2603)	Matched	0.3134	0.3214	−1.6	−0.34	0.7370

**Table 6:** The impact of national new areas on eco-efficiency: Other robustness.

Variable	3 Years in Advance	5 Years in Advance	7 Years in Advance	Reduce the Sample Size	Control Variables Lag by 1 Period	Control Variables Lag by 2 Period	Excluding Other Policy Effects
did	0.0376 **	0.0187	0.0111	0.0532 ***	0.0565 ***	0.0502 ***	0.0626 ***
	(0.0154)	(0.0178)	(0.0240)	(0.0150)	(0.0145)	(0.0147)	(0.0150)
National high-tech zones							0.0119 (0.0116)
advisl	−0.0127	−0.0137	−0.0137	−0.0174	0.0189	−0.0271	−0.0094
	(0.0188)	(0.0188)	(0.0188)	(0.0193)	(0.0188)	(0.0192)	(0.0188)
innovl	−0.0280 ***	−0.0281 ***	−0.0281 ***	−0.0144 ***	−0.0252 ***	−0.0185 ***	−0.0284 ***
	(0.0051)	(0.0051)	(0.0051)	(0.0056)	(0.0052)	(0.0054)	(0.0051)
openl	−0.2990 *	−0.2864 *	−0.2820 *	−0.0470	−0.3094 *	−0.1395	−0.3052 *
	(0.1707)	(0.1707)	(0.1707)	(0.1925)	(0.1654)	(0.1754)	(0.1706)
scienrsl	0.0558	0.0501	0.0461	−0.6676	0.0533	−0.5732	0.0428
	(0.4396)	(0.4399)	(0.4399)	(0.4348)	(0.4441)	(0.4864)	(0.4391)
govl	−0.0786 ***	−0.0797 ***	−0.0802 ***	−0.0528 ***	−0.0878 ***	−0.0779 ***	−0.0772 ***
	(0.0094)	(0.0094)	(0.0094)	(0.0109)	(0.0091)	(0.0093)	(0.0094)
_cons	0.4807 ***	0.4876 ***	0.4880 ***	0.4413 ***	0.2582 **	0.5131 ***	0.4611 ***
	(0.1188)	(0.1188)	(0.1188)	(0.1245)	(0.1197)	(0.1229)	(0.1187)
N	3948	3948	3948	3102	3666	3384	3948
R^2^	0.180	0.179	0.179	0.234	0.215	0.227	0.183

Notes: Robust standard errors are reported in parentheses; * *p* < 0.1, ** *p* < 0.05, and *** *p* < 0.01, respectively.

**Table 7:** Regression results of mechanism analysis.

	(1)	(2)	(3)	(4)	(5)
	*EE*	urbanl	*EE*	glhy	*EE*
did	0.0621 ***	0.0283 ***	0.0588 ***	0.4599 ***	0.0609 ***
	(0.0150)	(0.0072)	(0.0150)	(0.1598)	(0.0150)
urbanl			0.1172 ***		
			(0.0345)		
glhy					0.0026 *
					(0.0016)
advisl	−0.0100	0.0259 ***	−0.0130	−0.5066 **	−0.0087
	(0.0188)	(0.0090)	(0.0188)	(0.1995)	(0.0188)
innovl	−0.0284 ***	−0.0111 ***	−0.0271 ***	0.0645	−0.0285 ***
	(0.0051)	(0.0025)	(0.0051)	(0.0545)	(0.0051)
openl	−0.2950 *	−0.2622 ***	−0.2643	3.8882 **	−0.3052 *
	(0.1703)	(0.0817)	(0.1703)	(1.8108)	(0.1704)
scienrsl	0.0566	−0.2110	0.0813	0.5574	0.0551
	(0.4389)	(0.2106)	(0.4383)	(4.6662)	(0.4388)
govl	−0.0776 ***	−0.0105 **	−0.0764 ***	−0.1329	−0.0773 ***
	(0.0093)	(0.0045)	(0.0093)	(0.0994)	(0.0093)
_cons	0.4649 ***	0.2500 ***	0.4356 ***	3.3241 ***	0.4562 ***
	(0.1187)	(0.0570)	(0.1188)	(1.2616)	(0.1187)
N	3948	3948	3948	3948	3948
R^2^	0.182	0.219	0.185	0.063	0.183

Notes: Robust standard errors are reported in parentheses; * *p* < 0.1, ** *p* < 0.05, and *** *p* < 0.01, respectively.

**Table 8:** The results of regional heterogeneity test.

	(1)	(2)	(3)	(4)	(5)	(6)
	Eastern Region	Central Region	Western Region	Northeastern Region	Coastal Area	Inland Area
did	0.1392 ***	0.0552 **	−0.0056	0.0547	0.1266 ***	0.0230
	(0.0235)	(0.0276)	(0.0303)	(0.0444)	(0.0208)	(0.0202)
advisl	−0.0734 **	0.0610 **	−0.0664	0.0991 **	−0.0683 **	0.0244
	(0.0372)	(0.0280)	(0.0496)	(0.0418)	(0.0305)	(0.0244)
innovl	−0.0129	−0.0135 *	−0.0334 ***	0.0179	−0.0039	−0.0306 ***
	(0.0107)	(0.0075)	(0.0114)	(0.0191)	(0.0089)	(0.0064)
openl	0.6768 **	−0.0434	−1.2228 *	0.1405	0.4811 **	−0.3679
	(0.2777)	(0.2527)	(0.7161)	(0.3620)	(0.2090)	(0.2808)
scienrsl	3.3993 ***	−3.3589 ***	1.3875	5.3241 **	2.8515 ***	−0.5741
	(1.0208)	(0.4754)	(1.0957)	(2.3338)	(0.8981)	(0.5140)
govl	−0.1744 ***	0.0067	−0.0906 ***	−0.0966 ***	−0.1573 ***	−0.0601 ***
	(0.0273)	(0.0139)	(0.0192)	(0.0233)	(0.0234)	(0.0108)
_cons	0.7761 ***	−0.1331	0.8805 ***	−0.4822 *	0.6832 ***	0.2494
	(0.2395)	(0.1778)	(0.3041)	(0.2905)	(0.1972)	(0.1523)
N	1218	1120	1134	476	1414	2534
R^2^	0.358	0.233	0.115	0.287	0.365	0.125

Notes: Robust standard errors are reported in parentheses; * *p* < 0.1, ** *p* < 0.05, and *** *p* < 0.01, respectively.

**Table 9:** Results of layout model of national new areas and different types of cities.

Variables	(1)	(2)	(3)	(4)
	One New Area in One City	One New Area in Two Cities	Resource−Based Cities	Non-Resource-Based Cities
did	0.1187 ***	−0.0302	−0.0318	0.0585 ***
	(0.0189)	(0.0237)	(0.0492)	(0.0153)
advisl	−0.0107	−0.0148	−0.0027	-0.0297
	(0.0187)	(0.0188)	(0.0306)	(0.0241)
innovl	−0.0276 ***	−0.0279 ***	−0.0318 ***	−0.0228 ***
	(0.0051)	(0.0051)	(0.0075)	(0.0071)
openl	−0.2570	−0.2697	−0.8260 **	0.1318
	(0.1698)	(0.1709)	(0.3810)	(0.1887)
scienrsl	0.1108	0.0546	0.3802	−0.6351
	(0.4377)	(0.4399)	(0.6314)	(0.6323)
govl	−0.0773 ***	−0.0810 ***	−0.0648 ***	−0.0864 ***
	(0.0093)	(0.0094)	(0.0127)	(0.0146)
_cons	0.4644 ***	0.4940 ***	0.4330 **	0.5512 ***
	(0.1182)	(0.1189)	(0.1905)	(0.1536)
N	3948	3948	1582	2366
R^2^	0.187	0.179	0.135	0.237

Notes: Robust standard errors are reported in parentheses; ** *p* < 0.05, and *** *p* < 0.01, respectively.

## Data Availability

China City Statistical Yearbook (https://data.cnki.net/yearbook/Single/N2022040095 accessed on 6 June 2022); China Statistical Yearbook for Regional Economy (https://data.cnki.net/yearbook/Single/N2015070200 accessed on 10 July 2022); China Energy Statistical Yearbook (https://data.cnki.net/yearbook/Single/N2022060061 accessed on 10 July 2022); China Statistical Yearbook on Environment (https://data.cnki.net/yearbook/Single/N2022030234 accessed on 12 July 2022); Chinese Research Data Services Platform (https://www.cnrds.com/ accessed on 20 July 2022).
